# Exploiting Selective Position Labeling to Unveil the Hidden Complexity of Terminomics

**DOI:** 10.3390/molecules31071216

**Published:** 2026-04-07

**Authors:** Yuqing Deng, Minghao Li, Peicheng Lu, Bingbing Shi

**Affiliations:** 1 Department of Biochemistry and Molecular Biology, College of Basic Medicine, Jining Medical University, Jining 272067, China; dengmaggie81@gmail.com (Y.D.);; 2 Department of Chemistry and State Key Laboratory of Synthetic Chemistry, The University of Hong Kong, Pokfulam Road, Hong Kong SAR, China

**Keywords:** terminal modification, selective labeling, terminome analysis, protein profiling, mass spectrometry

## Abstract

Selective protein terminal labeling has become essential for system-wide studies of proteolytic mechanisms in disease. These methods enable precise tracking of cleavage dynamics, protease interactions, and cellular networks, offering transformative potential for proteolytic event analysis. This review explores recent advances in N-/C-terminal modification strategies, specifically for the applications in terminomics—the field focused on protein termini characterization. While protein termini provide valuable insights into functional proteome states, their low abundance in complex samples demands highly selective labeling approaches. We evaluate modern chemical and chemoenzymatic methods that leverage engineered chemical reactivity thresholds or enzymatic precision for site-specific modifications. Emerging strategies show enhanced substrate adaptability, reaction efficiency, and workflow compatibility, enabling broader applications in terminome studies.

## 1. Introduction

The capacity to selectively modify termini has laid the foundations for numerous breakthroughs in protease substrate profiling, disease biomarker discovery, and therapeutic target identification [1,2,3]. These site-selective strategies enhance the stability, detectability, and functional profiling of terminal peptides, which are critical for both fundamental and translational research innovations. These approaches enable precise functional manipulation while maintaining structural integrity of proteins. This precision facilitates the enrichment and labeling of terminal peptides, which are indispensable for identifying protease substrates across diverse biological samples, from cells to blood, and biopsy tissues [4]. Such advancements hold immense promise for mapping intracellular protease substrates; however, their application to membrane proteins is constrained by difficulties related to their low abundance [5]. Distinct membrane-bounded proteases may cleave the same protein at different sites, resulting in the generation of distinct functional protein forms with minor amino acid variations. Additionally, peptides at the N- and C-termini of proteins generally represent a minimal fraction within digested mixtures, thereby complicating the accurate identification of these proteoforms, referring to the different forms of a protein resulting from genetic variation, alternative splicing, truncations, or post-translational modifications (PTMs).

The termini of proteins or peptides have inherent accessibility with less structural limitations which offer great potential for site-selective labeling. The termini of proteins or peptides inherently offer structural accessibility with fewer spatial constraints, enabling site-selective labeling strategies [6,7]. While current approaches minimize perturbations from analogous functional groups in side chains [8,9], achieving broad adaptability across biological contexts remains challenging due to inefficiencies under physiological conditions [6,10]. Selective position labeling targeting specific residues (e.g., cysteine, lysine, or non-canonical amino acids) has emerged as a complementary strategy to traditional terminal modifications. Chemoselective reactions [10] and photoredox-catalyzed functionalizations [11] now enable residue-specific modifications without protective group strategies, overcoming limitations like competing nucleophilic sites [6,9]. The N-terminus remains advantageous due to its elevated nucleophilicity, allowing precise functionalization with reduced off-target effects. Most N-terminal protocols align with standard workflows, yet C-terminal labeling often requires elaborate protection–deprotection sequences due to its less distinctive reactivity [8]. Recent enzymatic ligation techniques [7] and transition-metal-mediated couplings [9] have expanded substrate scope for both terminal and non-terminal residues, enhancing therapeutic protein engineering applications.

Selective labeling focuses on the N-termini through its accessible α-amine group and the C-termini via its carboxyl group, holding the promise on an in-depth identification of terminal peptides within a biological system. Notably, terminomics is defined as the comprehensive study of biologically significant terminal peptide sequences in proteins, focusing on both native termini of mature proteins and proteolytically generated neo-termini. It represents a crucial frontier in functional proteomics, bridging protein structure analysis with dynamic proteolytic regulation through systematic terminal sequence characterization. These peptides, generated during proteolysis, function as dynamic regulators of cellular physiology, affecting signaling pathways and proteolytic cascades [2,12]. Considering the minimal fraction of termini in biological mixtures, several enrichment procedures are required to separately analysis by mass spectrometry. The labeling at termini directly collects terminal peptides, allowing simple and efficient workflow for the following analysis. Furthermore, selective labeling approaches improve ionization efficiency and optimize fragmentation patterns in mass spectrometry, significantly increasing the accuracy of peptide identification and quantification. These technical advancements are reshaping our understanding of proteolytic substrates and protein processing, unraveling previous opaque facets of cellular signaling pathways [13]. Although recent innovations in terminomic enrichment techniques have greatly expanded their utility in clinicals, the full spectrum of proteoforms generated during dynamic proteolytic events remains insufficiently explored [14]. Site-selective labeling approached address this gap by enabling the precise functional analysis of terminal modifications, facilitating deeper investigation of proteolytic events and their biological implications.

## 2. The Role of Protein Termini

**Figure 1:**
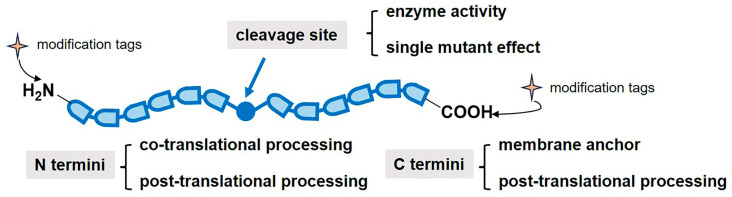
The role of protein termini and the generation of new protein termini in biological process. N-terminal isoforms may originate from alternative start codon usage and signal peptide removal during translation. Additionally, post-translational cleavage by proteases can generate new protein termini, sometimes unique to specific proteins or contexts, thereby altering function. Post-translational C-terminal cleavage may lead to loss of membrane association or interaction domains, marking a functional shift. The exposed regions of those protein termini are accessible to add functional groups or specific segments.

Proteins are initially synthesized with gene-encoded N- and C-termini, yet modifications and cleavage events can diversify their structure and function (Figure 1). Protein termini has elucidated their great value in maintaining metabolic stability of proteins and mediating protein degradation [15]. N-terminal isoforms may emerge from alternative translation initiation sites, methionine excision, and signal peptide removal during translation. At opposite termini, post-translational proteolytic cleavage of C-terminal regions can produce critical functional transformations. For example, loss of membrane association domains or interaction motifs may effectively reprogram protein localization and biochemical activities. These terminal domains serve as essential interaction hubs in biological systems, orchestrating the assembly of multiprotein complexes and regulating signal transduction cascades. Their structural architecture frequently incorporates conserved recognition motifs that mediate specific biomolecular interactions. A characteristic example involves the SRC homology 3 domain (or SH3 domain), which selectively bind proline-rich sequences preferentially positioned near terminal regions, illustrating how terminal domains coordinate intricate interaction networks [8]. Additionally, proteolytic terminal processing can prompt truncated forms with altered functional properties and stability profiles, often associated with pathological conditions, including cancer, neurodegenerative disorders, and cardiovascular diseases [2]. This enzymatic cleavage, through selective hydrolysis of peptide bonds, generates neo-N termini that allows proteins to transition between functional states or active certain biological pathways. From a chemical biology perspective, protein termini constitute dynamic regulatory platforms that integrate cellular homeostasis.

The exposed regions of those protein termini are accessible to add functional groups or specific segments. The study at termini not only emphasizes their essential roles but also exposes the current challenges in fully characterizing them, given their transient nature and the limited sensitivity of available analytical tools. Inspired by natural modification in biological processes, scientists have developed various labeling methodologies to depict dynamic systems and enhance analytical sensitivity.

## 3. Recent Advances in Selective Labeling Technique

### 3.1. Overview of Labeling Techniques

In biological systems, selective labeling techniques are essential for clarifying the intricate connections and activities of biomolecules. These approaches are distinguished by their capacity for precise tagging of protein termini, thus enhancing the accurate identification of molecular sites while reducing interference with natural biological processes. Thus, it offers an effective tool to obtain profound insights into biological mechanisms. Protein termini, including N- or C-termini of a polypeptide chain, serve as optimal sites for selective labeling owing to their unique properties and accessibility across diverse biological contexts. Labeling terminal regions with minimal steric hindrance preserves the protein’s natural structure and function, which is essential for studying dynamic biological processes with increased sensitivity and selectivity. These approaches alleviate interference with protein’s overall function or its interactions with other biomolecules, thereby facilitating a more precise investigation of protein roles in biological systems. Researchers can track protein localization, monitor conformational changes, and investigate interactions with other biomolecules [16,17]. The allure of specific terminal modification on native proteins or peptides is strong; however, challenges to achieve selective terminal modification are faced: the preference from flanking sequences, the compatibility with various types of amino acids at termini, reaction efficiency and kinetics in soluble buffer. Chemical conjugation and enzymatic tagging have been developed to specifically signify protein termini with probes. These breakthroughs might illuminate molecular-level biological processes by viewing and manipulating proteins in live cells, tissues, and organisms. A range of terminal labeling approaches will be covered in this chapter, detailing both the chemical and enzymatic methods involved. By comparing these strategies, we aim to highlight their transformative impact on protein biology and their potential to propel future discoveries in this field.

### 3.2. Chemical Labeling

**Figure 2:**
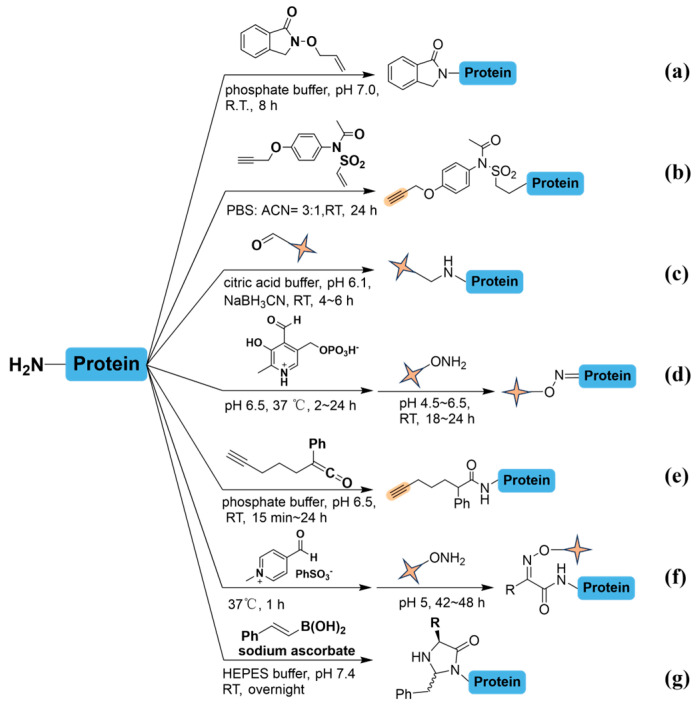
Traditional methods for selective terminal amine labeling to distinguish from other side chains. The quadrilateral star markers with pale-yellow color in the figure highlight key functional groups within the molecular structure. (**a**) Reactions were carried out in phosphate buffer (50 mM, pH 7.0) at room temperature (25 °C) for 8 h. (**b**) Incubation in PBS/ACN mixture (3:1, *v*/*v*) at room temperature (25 °C) for 24 h. (**c**) Reaction conducted in citric acid buffer (pH 6.1) containing sodium cyanoborohydride (0.1 M), maintained at 25 °C for 4–6 h. (**d**) Primary reaction is pH 6.5 at 37 °C for 2–24 h. Adjusted to pH 4.5–6.5 with hydroxyamine under ambient conditions for 18–24 h. (**e**) Short-term exposure in phosphate buffer (pH 6.5) at 25 °C for 15 min to 24 h. (**f**) Activation at 37 °C for 1 h and then hydroxyamine treatment at pH 5.0 under ambient conditions (25 °C) for 42–48 h. (**g**) overnight incubation (12–16 h) in 4-(2-hydroxyethyl)-1-piperazineethanesulfonic acid (HEPES) buffer (pH 7.4) under ambient conditions. The product is only formed when R=H under the used reaction conditions with proteins, despite the reaction is workable with a wide variety of amino acids in the N-terminal position (Val, Met, Trp, Cys, His, Ser, Tyr, and Arg).

Given the limited number of reactive groups naturally present and often repeated throughout protein or peptide sequences, achieving selective modification at specific termini has emerged as an essential strategy in protein and peptide engineering. The terminus presents inherent advantages for direct labeling, in contrast to side chains within sequences. However, traditional approaches often lack the required specificity, leading to heterogeneous products by off-target modifications. Traditional labeling approaches utilize solvent accessibility of protein termini and assess compatibility in aqueous buffers at room temperature (Figure 2). Notably, the protonation differences between the terminal α-amine (p*Ka* 6~8) and the lysine side chain’s aliphatic amine (p*Ka* ~10.5) provide a unique opportunity for selective modification approaches [18]. Leveraging this distinction, N-hydroxy-succinimide (NHS) derivatives under basic conditions have become a widely adopted method for modifying lysine residues. Initially, N-hydroxy-phthalimide (NHP) demonstrated specificity by selectively labeling the terminal amine of RNase A, bypassing intermediate lysine residues with high accuracy (Figure 2a) [19]. In physiological conditions, N-Phenyl-N-aceto-vinylsulfonamide has emerged as a potent agent for natural residue-specific modification of native peptides and proteins via aza-Micheal addition (Figure 2b) [20]. Additionally, biologically compatible imine condensation has been tested to optimize suitable structures containing aldehyde group (Figure 2c). A pH controlled reductive amination at N-termini preserved the positive terminal charge and offers a degree of selectivity [21]. Selective modification of the N-terminal α-amino group has also been achieved using oxone reagents, which transform it into an aldehyde or related forms suitable for conjugation with biophysical probes. Notably, Pyridoxal-5′-phosphate (PLP) was designed to convert N terminal amines into ketones or aldehydes, providing a site for single-position functionalization (Figure 2d) [22]. However, using high PLP concentrations or long reaction times may induce a side reaction that forms an N-terminal PLP aldol adduct and reduces labeling efficiency. Due to the presence of side reactions, this method is not suitable for labeling certain N-terminal residues (such as lysine and glutamine). In particular, PLP-mediated reactions involve imines or Schiff bases as intermediates, and their formation is thermodynamically reversible. Ultimately, in downstream LC-MS analysis, the incomplete and reversible nature of PLP-based transamination leads to systematic biases in quantitative results, increased missing data values, and complications in the interpretation and identification of complex spectra. Therefore, optimizing reaction conditions to improve conversion rates and carefully evaluating the effects of incomplete labeling during data analysis are critical to ensuring the successful labeling of a specific protein target. Additionally, an alkyne-functionalized ketene exhibited selectivity for 13 out of 20 natural terminal amino acids without oxidizing sensitive residues like cysteine, methionine, and tryptophan (Figure 2e) [23]. To address chemoselective alteration of all N-terminal amino acids, multiple oxidizing agents have been evaluated using XSKFR peptide sequences, with oxone (2KHSO_5_·KHSO_4_·K_2_SO_4_) identified as effective in generating oximes in tested peptides. This can then undergo transoximation with O-substituted hydroxylamines for further tagging [24]. Moreover, Rapoport’s salt (N-methylpyridinium-4-carboxaldehyde benzenesulfonate, RS) has been optimized for site-selective transamination at the terminal amine (Figure 2f) [25]. Activity assays have confirmed that RS-mediated alkoxyamine modifications don’t impair cellulolytic function. In parallel, vinylboronic acid has been explored for N-terminal functionalization in aqueous solution, circumventing traditional metal catalysts and minimizing off-target effects (Figure 2g) [26]. Nevertheless, the reaction parameters of these site-selective labeling reagents must be finely tuned into account for the diverse chemical and structural properties of different proteins.

**Figure 3:**
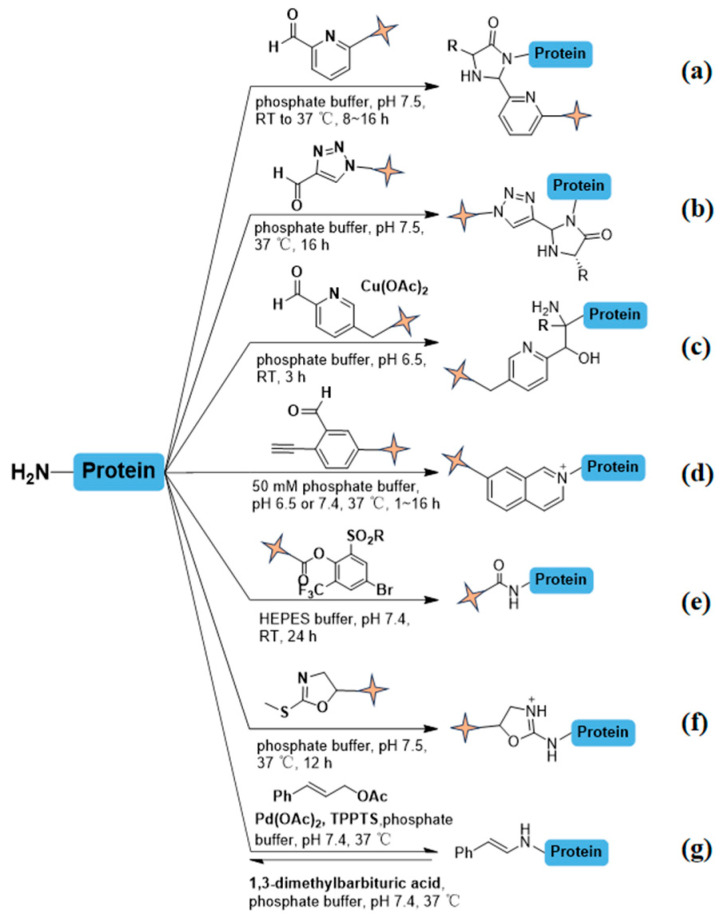
Advanced labeling techniques to accelerate selective modifications on N-termini. The quadrilateral star markers with pale-yellow color in the figure denote key functional groups within the molecular structure. Group R1 represents the side chain of first amino acid at N-termini. (**a**) Reaction conducted in phosphate buffer (pH 7.5) under temperature conditions ranging from room temperature to 37 °C, with a duration of 8–16 h. (**b**) Reaction performed in phosphate buffer (pH 7.5) at 37 °C for 16 h. (**c**) Reaction carried out in phosphate buffer (pH 6.5) at room temperature for 3 h. (**d**) Reaction executed in phosphate buffer (pH 7.5) at 37 °C, with a fixed duration of 12 h. The product is only formed when R=OH, NMe_2_. (**e**) Reaction utilizing 50 mM phosphate buffer (pH 6.5 or 7.4) at 37 °C, with variable reaction times spanning 1–16 h. (**f**) Reaction conducted in HEPES buffer (pH 7.4) at room temperature, maintained for 24 h. (**g**) Catalytic reaction system containing palladium(II) acetate ([Pd(OAc)_2_], 0.2 mM), trisodium triphenylphosphine tris(sulfonate) (TPPTS, 2.4 mM) in phosphate buffer (pH 7.4) at 37 °C. Reversible termination achieved via treatment with 1,3-dimethylbarbituric acid (5 mM) in phosphate buffer (pH 7.4) at 37 °C to regenerate natural termini.

Recently the specificity utility of 2-pyridinecarbaldehyde (2-PCA) and its analogues in conjugating biotin and fluorescent tags has underscored the value in functional and localization studies (Figure 3a) [27]. Following the formation of an imine intermediate via the aldehyde group and the N-terminal α-amino group, 2-PCA subsequently undergoes a cyclization reaction with the adjacent main chain amide group, resulting in the generation of a stable oxazolidine structure. This addresses the limitations of traditional Schiff’s base formation, characterized by inadequate product stability and the necessity for a subsequent reduction step. Involved in multiple lysines (ε-amino groups) of proteins, specific N-terminal modifications can be effectively achieved. The reaction typically occurs at physiological pH (7.0–8.5) and room temperature, thereby preserving protein conformation. Several 2-PCA derivatives have shown effectiveness for site-selective labeling on cell membrane [28] and intracellular delivery with minimal cytotoxicity, making them broadly compatible with complex contexts [29,30]. While 2-PCA analogues are versatile, they can encounter limitations, particularly when proline is present at the second position from N-termini. Cyclization with nitrogen on subsequent proline can’t form stable five-member rings. Moreover, the ε-amino group of the lysine residue does not react in this reaction due to the lack of a suitable neighboring amide group to participate in cyclization. A significant number of proteins feature proline precisely at the second position from the N-terminus in eukaryotic proteins. Therefore, the conventional 2-PCA N-terminomics may result in a systematic bias in the final N-terminal coverage, where Pro2 sites are generally underestimated or completely missed. This bias can affect the comprehensive understanding of specific proteolytic events, as many cellular proteases frequently generate new N-termini with Pro in the second position. Therefore, further development is needed to fully optimize 2-PCA as a general method for N-terminomics. The field continues to progress to one-step reaction, such as those using TA4C (1-(4-nitrophenyl)-1H-1,2,3-triazole-4-carbaldehyde) via Dimroth rearrangement (Figure 3b), which streamline bioconjugation and expand the scope of terminal labeling in biological systems [31]. Recently, a more efficient copper(II)-mediated aldol reaction has emerged (Figure 3c), further underscoring the ongoing innovation in this field and elucidating key aspects of proteomic dynamics [32]. Copper(II) simultaneously forms a five-membered ring chelate with the N-terminal amino group and the adjacent carbonyl oxygen, whereas the lysine side chain (ε-NH_2_) is excluded due to its inability to create a ring coordination configuration. It demonstrated high selectivity for N-terminal modification. At pH of 7.4, the reaction at room temperature shows a robust coordination capacity for the N-terminal α-NH_2_. The coordination-dominated lock-key recognition mechanism mitigates the constraints of the conventional dependence on p*Ka* variations, while the five-membered ring transition state lowers the activation entropy. To circumvent the aforementioned limitations associated with 2-PCA, 2-methyl thiol azolines has been introduced as a straightforward (Figure 3d), one-step labeling reagent for a wide range of peptides and proteins [33]. Furthermore, the hybridized nitrogen in azoline group enhances ionization, thereby increasing the mass spectrometric sensitivity of terminally modified peptides. Building on these findings, researchers have devised a practical approach to selectively modify the N-termini of proteins through a copper(II)-mediated [3+2] cycloaddition reaction with 2-PCA derivatives and commercially available maleimide derivatives under non-denaturing conditions [34].

Bio-orthogonal reactions utilizing aldehyde groups have also gained popularity. For example, 2-ethylbenzaldehyde (2-EBA) is compatible with diverse functional groups (Figure 3e), including alkynes and fluorescein [35]. These explorations in terminal amine modifications are not only expanding our toolkit for investigating protein function but also refining our ability to probe protein behavior within complex biological environments. Additionally, a novel class of phenol esters has been engineered for N-terminal acylation of biomolecules, where the ortho-positioned sulfonic acid or sulfonamide groups provide steric protection against hydrolysis (Figure 3f) [36]. Despite these advances, developing simple and versatile methods for diverse biological applications remain challenging. Recent breakthroughs in palladium catalysis and Mo(CO)_6_ as a carbonyl source have pioneered γ-C(*sp3*)-H activation, enabling selective functionalization of aliphatic side chains in unprotected N-terminal peptides [37]. Remarkably, residues such as valine, isoleucine, and L-tert-leucine have shown high conversion rates, which is uncommon in other selective methods. Additionally, a reversible palladium-catalyzed approach has proven compatible with all 20 canonical amino acids under physiological conditions (Figure 3g). In the conjugation phase, Pd(0) catalysts facilitate selective transfer of cinnamyl groups to the α-amino group of N-termini under physiological conditions. This occurs via oxidative addition of cinnamyl carbonate to Pd, followed by 1,3-dimethylbarbituric acid (DMBA) as a Pd-binding agent induces retro-allylation. This triggers β-hydride elimination at the modified N-terminus, regenerating native peptides/proteins through Pd(II)-mediated β-oxygen eliminationamine coordination and transmetalation to form a stable N-cinnamyl intermediate. The reversibility allows transient N-terminal tagging with azide handles for a cleavable biotinylated linker via click chemistry. After digestion by trypsin, N-terminal modified peptides were enriched by streptavidin agarose and following formic acid treatment release N-terminal peptides for mass analysis. This provides detailed studies of N-terminal proteins and facilitates traceless enrichment of proteins for N-terminome analysis [38].

**Figure 4:**
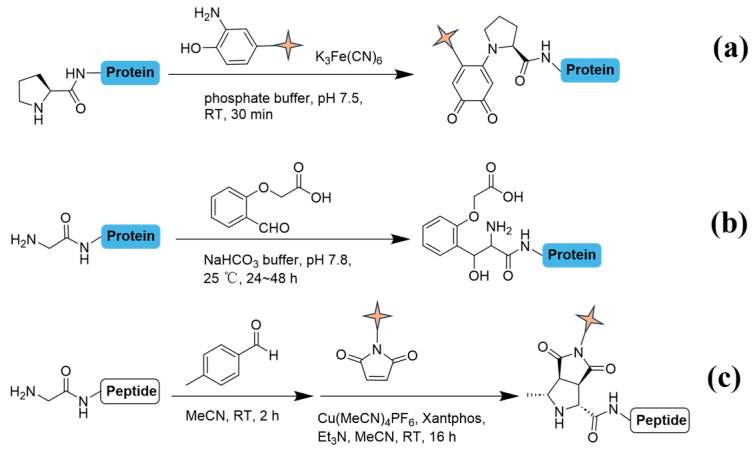
Specific labeling methods for proline and glycine on N-termini. (**a**) Reaction in phosphate buffer (0.1 M, pH 7.5) with K_3_[Fe(CN)_6_] (5 mol%) at 25 °C for 30 min. (**b**) NaHCO_3_ buffer (50 mM, pH 7.8) at 25 °C for 24–48 h. (**c**) At 25 °C for 2 h, then sequential addition of [Cu(MeCN)_4_]PF_6_ (0.1 equiv), Xantphos (10 mol%), Et_3_N (3 equiv) in MeCN at 25 °C with stirring for 16 h. Pale-yellow quadrilateral star markers highlight key functional groups.

The specific amino acids at N-termini exhibit distinct characteristics that offer potential for site-selective conjugation (Figure 4). One approach entails the oxidation of o-aminophenols using potassium ferricyanide (Figure 4a), which accelerates their conjugation with terminal proline via rapid second-order kinetics [39]. The followed conjugation with hydrazine or alkoxyamine effectively distinguishes of proline from other proteinogenic amino acids [40]. For N-terminal glycine, covalent strategies also exist; an aldehyde, optimized with hydrogen bond acceptor forms a stable amino alcohol with terminal glycine. The application of 2-(2-formylphenoxy)acetic acid and its derivatives have displayed significant selectivity and efficiency in physiological conditions (Figure 4b) [41]. Recently, a mild copper-catalyzed three-component [3+2] cycloaddition has offered a versatile method for installing two distinct functional groups at terminal glycine (Figure 4c), accommodating up to 26 amino acid residues [42]. Additionally, the N terminal cystine site supports diverse conjugation methods reported which are not discussed here for terminomics [43,44].

**Figure 5:**
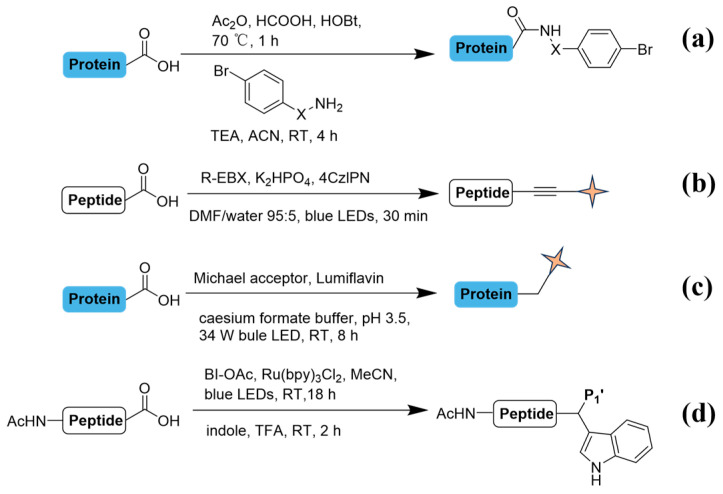
Chemical labeling methods on C-termini. For reaction conditions: (**a**) Ac_2_O (3.0 equiv), HCOOH (2.0 equiv), HOBt (0.2 equiv), 70 °C, 1 h (argon atmosphere), 4-Bromoaniline was added and reacted for over 12 h. (**b**) NaHCO_3_ buffer (0.1 M, pH 7.8), 25 °C, 24–48 h. (**c**) 4CzIPN (30 mol%), K_2_HPO_4_ (10 equiv) in DMF/water 95:5 (10 mM) at 25 °C for 30 min. 4CzIPN represents 2,4,5,6-tetra(9H-carbazol-9-yl)isophthalonitrile. (**d**) BI-OAc (3.0 equiv), Ru(bpy)_3_Cl_2_ (30 mol%), MeCN, blue LEDs, room temperature, 18 h, then indole (2.0 equiv), TFA (15 equiv) added, room temperature, 2 h. BI-OAc represents acetoxybenziodoxolone. (**a**) X = CH_2_CH_2_, NH; (**b**) X can be any electron withdrawing group such as cyano, keto, or ester groups; R1 group represents structures with a functional group. R2 group represents benzene derivatives with a functional group. The R3 moiety corresponds to the variable side chain of the C-terminus amino acid in the peptide sequence. Pale-yellow quadrilateral star markers highlight key functional groups.

Techniques for C-terminal modification have been relatively less explored considering the obscure reactivity with other carboxylic groups in sides chains. The development of chemoselectivity at C-termini remains in its nascent stages and has significant progress yet to be achieved (Figure 5). Oxazolone-based derivatization was historically the first commonly used chemical modification for C-termini (Figure 5a) [45]. It hasn’t been widely applied, mainly limited by its low reactivity, despite several derivative reagents being reported for improved modification and enrichment [46,47]. Nucleophilic residues such as cysteine thiols, lysine amines, and histidine imidazoles can potentially react with intermediate electrophilic species formed during the alkylation process. These side reactions may compromise selectivity for the intended C-terminal modification. Additionally, aromatic amino acid side chains (e.g., tryptophan, tyrosine) may undergo radical chain transfer, which could lead to undesired functionalization. This phenomenon highlights the need for carefully optimized reaction conditions to minimize off-target modifications. These inherent limitations have severely compromised the reliability and quantitative accuracy of the modification. Consequently, although oxazolone-based derivatization holds historical significance, the challenges it faces in practical applications have prevented it from becoming a mainstream choice in current high-throughput and high-confidence strategies for C-terminal peptide enrichment and identification. Recently, methods like visible-light-mediated decarboxylative alkylation have demonstrated potential for site-selective modification (Figure 5b) [48]. The selectivity relies on the difference in the oxidation potentials between carboxylates on amino acid side chains and the C-terminus. Glutamate (Glu) and aspartate (Asp) side-chain carboxylates (p*Ka* ~4) may compete with the C-terminal carboxyl group (p*Ka* 2–3) under non-ideal pH conditions. This competition could diminish the regioselectivity observed in experiments featuring optimized reaction parameters. It is noteworthy that different C-terminal residues exhibit significant differences during the conversion process. Among them, aspartic acid (Asp)/glutamic acid (Glu) maintain high selectivity (yield > 75% at pH 7), while lysine (Lys)/tyrosine (Tyr)/histidine (His) require adjusting the pH to 3.5 or using a milder photocatalyst to achieve efficient reaction. The stable heteroatom α-amino radical at protein termini has instinct advantages to site-selective labeling for endogenous peptides and protein insulin. A broad tolerance for different C-terminal amino acids was also elucidated under mild physical circumstances, albeit with inconsistent conversion rates. Additionally, a direct and selective labeling method for C-terminal peptides was proposed, eliminating the need for hazardous catalysts. Under mild LED illumination, a swift and efficient conjugation at C-termini was optimized, providing a metal-free decarboxylative alkynylated handle and exhibiting good tolerances for various reactive side chains (Figure 5c). To uncover short peptides up to tetramer, intermediates like N,O-acetals was formed at C-termini via visible light, leading to novel peptide scaffolds and bioconjugation (Figure 5d) [49]. Structural diversification via β-carbon hydrogen elimination could lead to alkene byproducts. Transition-metal-based photocatalysts, such as Ru(bpy)_3_^2+^, may also directly oxidize electron-rich residues under certain conditions. While the spatial confinement of radical generation to C-terminal residues (Figure 5b–d) inherently suppresses many off-target reactions, concerted optimization of illumination parameters, photocatalyst selection, and reaction medium remains essential. C-terminal modifications are gaining momentum, with expectations for more innovations to address current challenges. Although labeling techniques for C-termini are fewer compared to those for N-termini, there is still significant potential to explore novel approaches for C-termini labeling.

The essential difference between cleavage-based modification and direct C-terminal modification resides in their underlying mechanisms and resulting effects. Direct modification utilizes chemical strategies, such as oxazolone derivatization or visible-light-mediated alkylation, to selectively target the terminal carboxyl group while preserving the integrity of the polypeptide chain. This method emphasizes the importance of maintaining protein integrity while encountering difficulties in differentiating between C-terminal and side-chain carboxyl groups. In contrast, cleavage-based procedures like carboxypeptidase Y digestion or sequence-specific proteases fragment the protein enzymatically or chemically at predefined sites, providing new termini for modification. This technique improves selectivity through cleavage-site recognition, but it modifies protein structure, limiting its use in functional research. Nonetheless, it proves advantageous for analytical workflows, such as mass spectrometry-based proteomics, where fragmentation facilitates sequence identification. Direct methods are favored for therapeutic protein engineering that necessitates intact structures, whereas cleavage-based strategies are particularly effective in peptide mapping, terminal sequencing, and the analysis of post-translational modifications. The choice of method depends on experimental priorities for protein integrity against thorough chemical characterization. Both methods encounter trade-offs between structural integrity and analytical precision.

### 3.3. Chemoenzymatic Labeling

**Figure 6:**
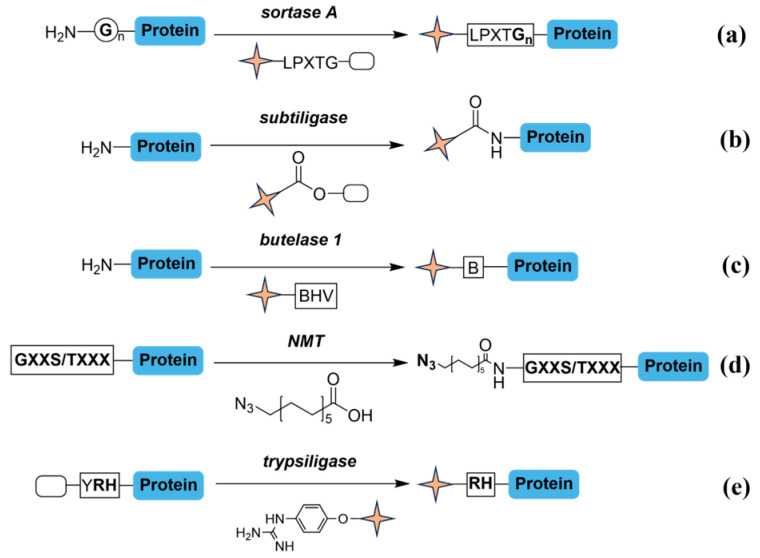
Enzymatic labeling methods on N-termini. (**a**) protein substrate (60 µM), sortase A stock (6 µM, 0.1 mole equivalents), depsipeptide stock (120 µM, 2 equivalents) and ligation buffer, 37 °C, 4–6 h. (**b**) 1 µM subtiligase, 5 mM donor peptide ester, 50 µM protein substrate, room temperature, 1–2 h. (**c**) 100 µM protein substrate, one or two molar equivalents of thiodepsipeptide, and 50 nM of butelase 1 (0.0005 molar equivalent) in 1 mM EDTA, 20 mM phosphate buffer (pH 6.5), 42 °C. B represents aspartate or asparagine. (**d**) 500 µM 12-Azidododecanoic acid (12-ADA), 1 mM IPTG, 37 °C, 3–4 h. (**e**) 100 mM substrate, 10 mM trypsiligase in 50 mM ZnCl_2_, 100 mM HEPES, 100 mM NaCl, 10 mM CaCl_2_ (pH 7.8), 1 h, then a threefold excess of 4-OGp was added and incubated for 1 h. Pale-yellow quadrilateral star markers highlight key functional groups.

Modifications at the N- or C-termini of proteins through targeted enzymatic reactions are essential in protein engineering, structural biology, and therapeutic applications [50]. These modifications, frequently involving precise enzymatic ligations, enable site-selective protein bioconjugation and have led to the development of various techniques aimed at improving accessibility and adaptability (Figure 6) [51]. Sortase A, a prevalent enzyme from Staphylococcus aureus, recognizes the LPXTG peptide sequence (with X being any amino acid). Sortase A cleaves between the threonine (T) and glycine (G) residues, facilitating the attachment of the terminal glycine sequence to the N-termini of another protein (Figure 6a) [52]. Direct evolution has further optimized the multifunctionality and catalytic capacity of sortase, allowing for modification across various peptides or proteins of different lengths [53]. This precision in labeling and the enzyme’s adaptability to different substrates have rendered it exceptionally useful for terminal modifications [54,55,56,57]. Unfortunately, sortase A encounters constrains, particularly regarding its activity and stability. Reversible reactions caused by nucleophilic side-products can occasionally impede its applications [58].

Subtiligase, a peptide ligase derived from the serine protease subtilisin of *B. amyloliquefaciens*, catalyzes ligation between a peptide ester acyl donor and the N-terminal α-amine of a peptide or protein (Figure 6b) [59,60]. The engineered subtiligase variants have great efficiency and site specificity; nevertheless, their ligation activity is biased due to significant substrate preferences at the P1′ and P2′ positions. Small amino acids are favored at P1′, while aromatic and large hydrophobic residues are preferred at P2′, leading to incomplete ligation and labeling bias, particularly in complex samples. The reaction conditions are mild, maintaining the structural integrity and functionality of the implicated proteins, and subtiligase is especially beneficial for chemo-proteomic profiling of cellular N-termini [61,62,63,64,65]. Nevertheless, specific complex proteins pose difficulties for subtiligase, mostly due to steric hindrance or folding constraints, which can be alleviated through meticulous substrate selection or engineering [59]. A combination of subtiligase variants now allows for the efficient labelling and enrichment of diverse protein N-termini [66].

**Figure 7:**
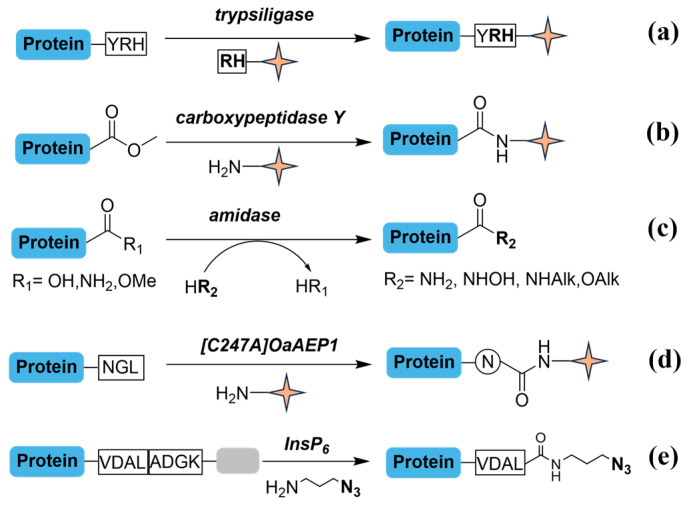
Enzymatic labeling methods on C-termini. Pale-yellow quadrilateral star markers highlight key functional groups. (**a**) 100 mM protein substrate, 1 mM Arg-His substrate, 10 mM trypsiligase, 100 mM HEPES/NaOH pH 7.8, 0.1 mM ZnCl_2_, 100 mM NaCl, 10 mM CaCl_2_, 20 °C, 1 h. (**b**) 1 mg/mL methyl esterified protein substrate, 150 mM biocytinamide, and 0.2 mg/mL carboxypeptidase Y in 0.1 mM sodium bicarbonate, pH 11.6, 0.1% SDS, 1% Triton-X 100, 37 °C, 30 min. (**c**) 5 mM protein substrate, 1.4 mg/mL peptide amidase, and various amine nucleophiles in acetonitrile, 50 °C, 1 day. (**d**) 100 µM protein substrate, 1 µM [C247A]OaAEP1 (0.01 equiv), and 100 mM amine necleophile in PBS, pH 7.4, 25 °C, 4–6 h. (**e**) 50–100 µM CPD-tagged protein substrate, 5 µM InsP_6_, 5 mM amines, and 5 mM DMAP in 50 mM NaOAc buffer pH 8.0, 4 °C, 3 h. The grey color represents the cysteine protease domain (CPD) of the protein substrate.

Butelase-1, an asparaginyl ligase originating from *Clitoria ternatea*, is renowned for its remarkable catalytic efficiency, achieving rates of up to 542,000 M^−1^s^−1^ [67]. It is extensively utilized in protein and peptide engineering, enabling high-yield, irreversible linkages at low substrate and concentrations (Figure 6c) [68]. The reaction process relies on the thiol group of the substrates as a guiding entity, promoting efficient ligation. It recognizes an Asn/Asp-HV motif at the C-terminus of a donor substrate, releasing the HV dipeptide and forming an Asn-enzyme thioester intermediate. This intermediate is then attacked by the N-terminus of a target acceptor protein. N-myristoyl transferase (NMT) identifies a flexible short peptide motif at protein N-termini, generally (M)GXXXS/TXXX, and bio-orthogonally transfers azide-modified myristate analogs to these proteins under cellular conditions (Figure 6d) [69]. Trypsiligase, a form of trypsin, notably cleaves the tripeptide motif Y-RH, thereby allowing rapid transpeptidation of the acyl structure to an α-amine carrying an N-terminal RH motif (Figure 6e). Under optimal conditions, mutant trypsiligase selectively modifies N-terminal residues in diverse proteins [70], as well as the C-termini of specific antibody fragments, such as Her2-specific Fab [71] (Figure 7a). Moreover, site-selective labeling at both N- and C-terminal of peptides and proteins has been employed to incorporate clickable anchors into the human protein cyclophilin 18 and the antibody Fab fragments anti-TNF-α and anti-Her2 [72].

Subtiligase has the ability to specifically label native N-termini, but its application scope is limited by its stringent requirements for both the P1′ and P2′ sites. Sortase A recognizes the C-terminal motif (LPXTG), which grants it high site specificity. However, the reaction is reversible and its efficiency is relatively low. Butelase-1 is currently one of the most efficient peptide ligases known. Yet, its substrate recognition is also extremely stringent, potentially making it suitable only for substrates with specific C-terminal sequences (Asn/Asp-HV). Each enzyme is a powerful tool in proteomics, but each has its unique properties or limitations due to differences in substrate specificity or modification sites. When applied to global terminomics analysis, they may introduce non-negligible quantitative bias. Therefore, it is advisable to weigh the pros and cons of each enzyme before deciding which one to use. Nonetheless, subtiligase demonstrated greater substrate versatility and catalytic efficiency than sortase A and butelase-1 [66].

To tackle selective positioning at the C-termini, several enzymatic techniques have been reported to transform the terminal site into more reactive configurations for subsequent tagging (Figure 7). Carboxypeptidase Y, for instance, removes amino acids from the C-termini and has shown the compatibility with chemical labeling for newly exposed carboxyl groups (Figure 7b). The metal-free catalyst addition allows carboxypeptidase Y is compatible for endogenous labeling, paving the way for direct modification of C terminome [73]. Furthermore, a peptide amidase engineered from *Stenotrophomonas maltophilia* efficiently converts carboxyl groups into a nonaqueous environment (Figure 7c). The rational protein design provides enhanced robustness for harsh terminal reaction circumstances, giving its potential for selective C-terminal functionalization [74]. Another enzyme, a promiscuous transpeptidase [C247A]OaAEP1, has demonstrated the ability to irreversibly modify C-terminal asparagine residues (Figure 7d), turning it appropriate for protein-drug conjugate [75]. A recent advancement employs InsP_6_-triggered cysteine protease domains for self-cleavage, promoting functionalization and de-functionalization of protein C-termini with a wide range of amines (Figure 7e) [76].

Enzymes that participate in protein ligation and labeling capitalize on their inherent specificities, facilitating selective, covalent modifications of protein termini. The precise labeling in extracellular matrix provides spatial localization and structural integrity, indicating connections within complex biological networks. The exceptional chemo- and regioselectivity of enzymes makes them invaluable tools for precise terminal modification in intricate processes, allowing for the snapshot of dynamic proteolysis.

### 3.4. Specificity and Efficiency

Site selectivity in terminal modification refers to the precise modification of reactive terminal regions, specifically the N- or C-termini, without affecting internal amino acid residues. This selective targeting leverages the distinct microenvironments, setting them apart from the rest of the protein structure [77,78]. High labeling efficiency is crucial for reliable mass spectrometry-based quantifications [79]. Incomplete labeling generates high noisy signals and obscures low-abundance peptides. Achieving efficient and consistent terminal labeling thus improves data clarity and supports accurate protein identification through enhanced sequence mapping. For instance, the reagent 4-(guanidinomethyl)-benzoic acid (Gmb) has proven effective for N-terminal modification, facilitating peptide fragmentation and improving mass spectrum interpretation [80]. Specifically, the success of terminal modification is influenced by multiple factors, including substrate sequences, cellular conditions, and structural accessibility. Consistent terminal modification across mixed samples strengthens proteome quantification and can improve proteins stability, as demonstrated with several N-terminal selective reagents [81]. C-terminal modification, as critical as N-terminal modification, comes with its own unique challenges for delicate labeling at physiological condition. The attraction for enzymatic catalyzed modifications at C-termini are obtained for highly selective labeling to a wide range of substrates. Ensuring accessibility of terminal sites while minimizing disruption with protein function is crucial for preserving the biological relevance of these modifications. Despite the evolving developments for novel reagents or catalysts around the termini, faster reactions, higher yields, and minimized side reactions are essential for producing modified proteins in high throughput manner.

## 4. Terminal Modification Applied in N-Terminomics

As detailed in Section 3, selective labeling strategies are pivotal for targeting and enriching protein termini. These approaches, whether based on chemical principles (e.g., NHS esters, 2-PCA, oxime chemistry) or enzymatic mechanisms (e.g., Subtiligase, Sortase A), aim to achieve efficient and specific modification of terminal peptides, thereby providing a “handle” for subsequent capture and mass spectrometric analysis. This chapter focuses on how these labeling strategies are integrated into specific terminomics workflows, including enrichment strategies (positive and negative) and their applications in complex biological samples. By highlighting concrete examples, we demonstrate the transformative potential of these techniques in advancing our understanding of protein biology and its implications in health and disease.

**Figure 8:**
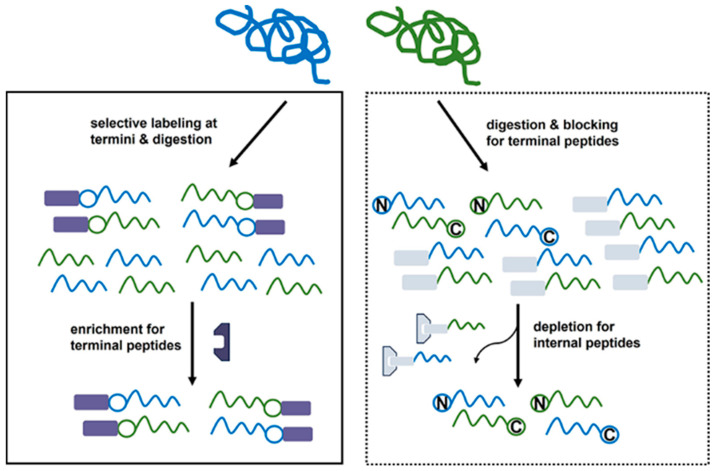
The workflow illustrates both positive (**left**, solid frame) and negative (**right**, grid frame) enrichment strategies for isolating protein termini. Firstly, target proteins were extracted from complexed samples. In the positive enrichment approach (**left**, solid frame), protein termini are selectively labeled, followed by digestion into peptide mixtures. These modified terminal peptides are then directly isolated using affinity-based chromatographic methods. In the negative enrichment approach (**right**, grid frame), all reactive groups in protein mixtures are initially labeled, typically via acetylation or dimethylation. After digestion, usually with trypsin or Lys-C, internal peptides are selectively removed through additional chemical blocking, which differentiates them from terminal peptides. This process allows terminal peptides to be separated and collected in mobile phase.

The term “terminomics” originated from proteomics as an extension that encompasses the full spectrum of protein stasis and functional activities related to protein termini. The proteoforms in dynamic biological processes may not consistently present balanced or intact patterns and the nuances may be dismissed in complicated contexts, particularly in low abundance. Consequently, it is imperative for terminal enrichment to ensure the capture and identification of critical changes or cleaved forms of proteins captured and identified. To address this issue, several enrichment techniques, both positive and negative, have been developed to focus on the enrichment and identification of protein termini (Figure 8) [82,83,84]. In practice, target proteins were firstly extracted from complexed samples. In the positive enrichment technique, protein termini are specifically labelled, subsequently followed by digestion into peptide mixtures. These modified terminal peptides are then directly isolated using affinity-based or chromatographic methods prior to identification. While naturally modified termini are excluded, positive enrichment techniques have increased the sensitivity for inherent unmodified termini and neo-N or C termini by proteolysis. The negative enrichment technique circumvents this issue by blocking all reactive groups in protein mixtures and removal of internal peptides. The detailed separation methods have been carefully reviewed. Recent studies have shown that non-enrichment N-terminomics strategies based on peptide fractionation techniques (such as FAIMS [85,86] and bRP [86]) hold significant potential, alongside traditional positive and negative enrichment methods for the identification of protein terminomes. In this chapter, we will delve into specific examples that illustrate the application of selective labeling of protein and peptide termini in terminomics (Table 1). By highlighting these, we seek to demonstrate the transformative potential of these techniques in advancing our understanding of protein biology and its ramifications in health and illness. This exploration aims to inspire further innovations that will continue to propel the field of terminomics forward.

### 4.1. Positive Enrichment Strategies for N-Terminomics

#### 4.1.1. Chemical Modification

**Figure 9:**
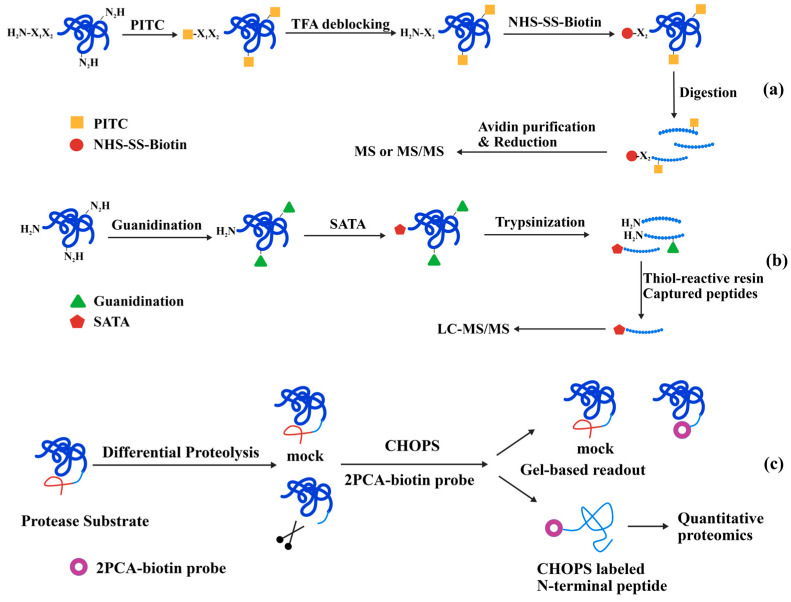
Workflow for chemical modification of terminal identification: (**a**) N-CLAP; (**b**) SATA-based resin-assisted enrichment method; (**c**) chemical enrichment of protease substrates (CHOPS).

Selective chemical approaches have significantly encouraged the investigation of protein termini by enabling more targeted reactions with the free amine or carboxyl groups of terminal amino acids. These methods have expanded the edge of terminome analysis, offering greater sensitivity and selectivity in detecting terminal peptides. Furthermore, these chemical handles have streamlined methods that integrate mass spectrometry, expediting extensive investigations and offering comprehensive insights into the structural and functional landscape of proteomes. The Edman degradation, an early instance of selective chemical modification involves the blocking of all amines in a protein by phenyl isothiocyanate (PITC). Trifluoroacetic acid was subsequently used to selectively remove the N-terminal protecting groups, preserving the PITC-treated lysine. This principle was implemented in N-CLAP (N-terminome by chemical labeling of the α-amine of proteins), enabling the detection of 278 N-terminal peptides and revealing proteolytic cleavage events by signal peptidases (Figure 9a) [87]. A distinct approach has entailed selective guanidination at lysine site, succeeded by conjugation of N-succinimidyl S-acetylthioacetate (SATA) to the terminal amine, a procedure shown to be nearly quantitative. The analysis of the cell lysate revealed 1672 unique protein N-termini or proteolytic cleavage sites from 690 individual proteins (Figure 9b) [88]. The biotinylated reagent PFP (pentafluorophenyl)-Rink-biotin has also been utilized, reacting with terminal amines following protein guanidination to produce a signature ion in mass spectrometry, facilitating the identification of N-terminome [89].

The 2-pyridinecarboxaldehyde (2-PCA) derivative (2PCA-biotin), a representative N-terminal selective labeling reagent introduced in Section 3.2, has been developed to overcome the sequence constraints in unbiased protease substrate profiling with quantitative proteomics, known as chemical enrichment of protease substrates (CHOPS) (Figure 9c) [90]. More recently, a version of 2PCA-biotin derivative featuring a cleavable linker was reported to undergo aldol condensation with N-termini in a study concerning colorectal cancer and tumor-adjacent tissues. This approach identified 4686 unique N-terminal peptides, indicating the potential role of abnormal protein hydrolysis in the pathogenesis of colorectal cancer. Notably, it overcomes the Pro2 limitation, enabling more comprehensive N-terminome coverage [91]. Furthermore, an alkyne-modified derivative of 2PCA, a commercially available reagent, has been applied in the proteomic profiling of protease cleavage sites, defining rapid and global sequencing of apoptotic proteolytic events [92]. Moreover, the integration of 2PCA with terminal amine capture and biotinylation of lysine side chains has proven effective in enriching C-terminal residues followed LysC digestion. This method facilitates efficient and high-throughput analysis of the protein C-terminome [93].

#### 4.1.2. Chemoenzymatic Labeling Techniques

**Figure 10:**
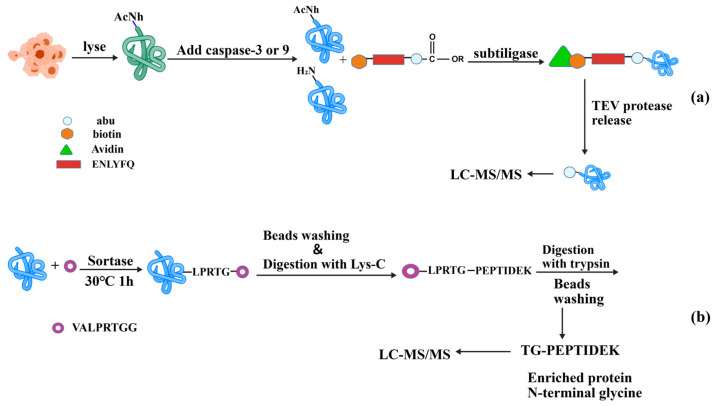
Workflows for chemoenzymatic labeling-based terminal identification. (**a**) Subtiligase-mediated biotinylation; (**b**) Sortase A-mediated chemoenzymatic enrichment.

Recent progress in enzymatic engineering has expanded the capacity to study protein terminus with increased specificity and sensitivity. The evolution of enzymatic engineering allows for the meticulous regulation of catalytic activity, adaptation for substrate diversity and modification of reaction conditions. The use of chemoenzymatic labeling in terminomics identification has provided systematic investigations of dynamic processes at termini, revealing cellular homeostasis and regulation. Subtiligase, as a representative utilized in terminomics, demonstrates broad substrate compatibility with acyl donors and acceptors. It labels α-amines using a peptide ester that comprises a biotin tag, a TEV-protease cleavage site, and an amino-butyric acid mass tag. After enzymatic ligation, biological samples are generally subjected to digestion, after which the ligated termini are captured by avidin beads and subsequently released via TEV protease hydrolysis. Subtiligase-mediated N-terminal labeling has been successfully employed to profile caspase-3 and caspase-9 substrates (Figure 10a) [94], and identify host cell substrates targeted by SARS-CoV-2 proteases Mpro and PLpro [64]. Additionally, this technique was adopted for upregulation associated with disease mechanism studies such as ischemia reperfusion [95] and prion disease pathogenesis [96]. Alternatively, a mutant peptide ligase known as stabliligase, engineered with an N-terminal nucleophile, entitled covalent attachment to cell surface glycans when paired with sodium periodate. This adaptable method has been exploited across multiple cell lines, including primary immune cells, to uncover major cleavage sites in extracellular proteomics [97]. Sortase A, another enzyme detailed in Section 3.3, specifically recognizes the LPXTG motif and transfers a moiety to an N-terminal glycine. This chemoenzymatic approach has been used to enrich and capture proteins with N-terminal glycine, revealing its impact on protein stability (Figure 10b) [98].

### 4.2. Negative Enrichment Strategies for N-Terminomics

**Figure 11:**
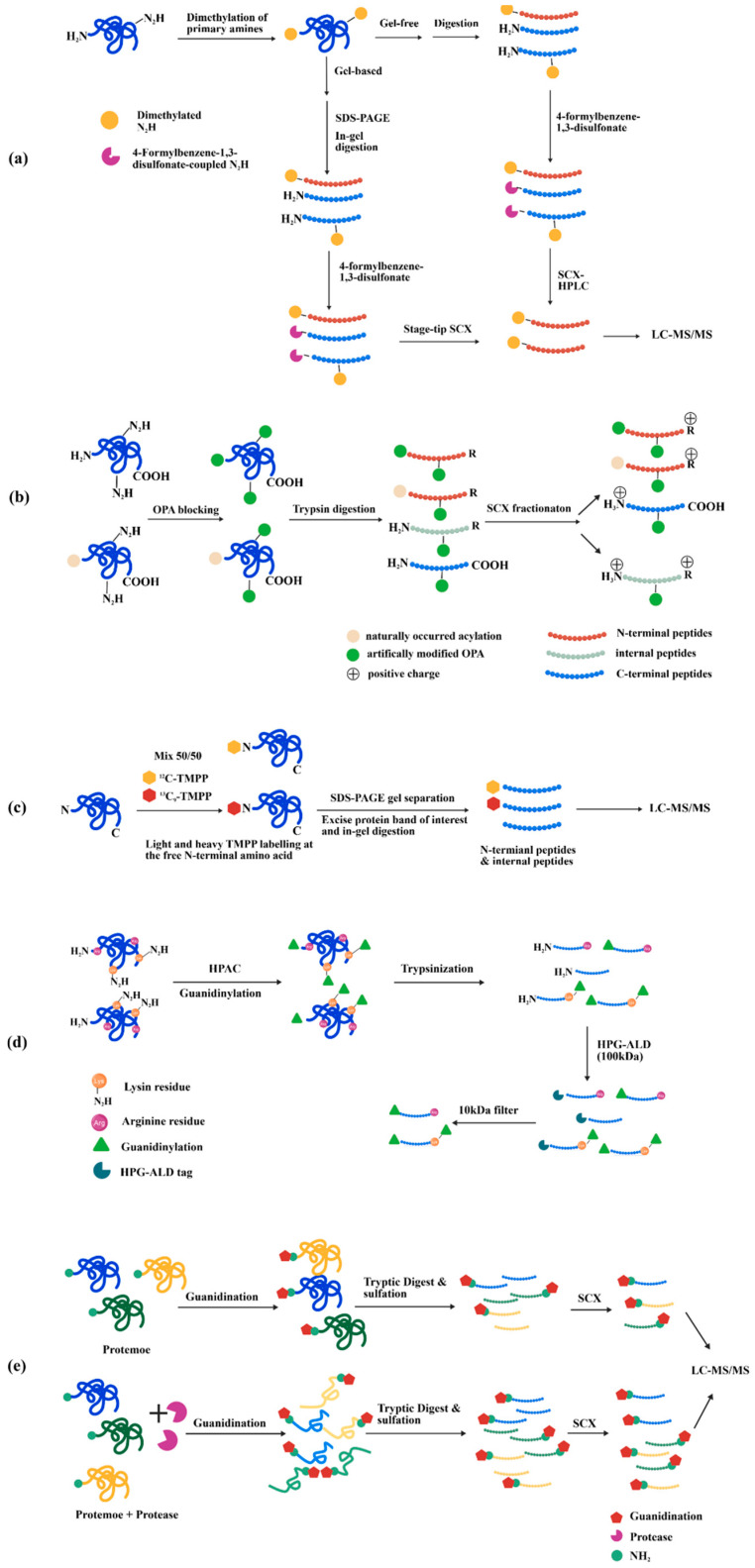
Workflow for negative enrichment strategies. (**a**) Charge reversal combined with SCX enrichment; (**b**) OPA labeling-SCX fractionation; (**c**) TMPP-labeled dN-TOP approach (**d**) TAGS; (**e**) TAGS-CR.

Recent developments encompass the High-efficiency Undecanal-based N-Termini EnRichment (HUNTER) technique, which utilizes peptide α-amine and undecanal in 40% ethanol to reveal system-wide N-terminal profiles from minute samples [99]. Sodium 4-formylbenzene-1,3-disulfonate is employed to invert the charge of internal peptides, hence prevent interference from newly generated neo-N termini during digestion. A straightforward N-terminomic strategy was applied to investigating the N-terminome of mouse embryonic fibroblasts cells deficient in both cathepsin B and L, following separation via strong cation exchange chromatography (SCX), compared to wild type controls (Figure 11a) [100]. SCX-based enrichment of N-terminal peptides relies on a charge difference under low pH conditions. At acidic pH, internal peptides possess a free α-amine, which provides an additional positive charge compared to their N-terminally blocked counterparts. This charge difference causes internal peptides to bind more strongly to the SCX resin, while N-terminally blocked peptides, with lower affinity, are eluted first. Besides, Traut’s reagent has been successfully integrated with gold nanoparticles [101] or Fe_3_O_4_ nanoparticles [102] to establish straightforward and efficient procedures for identifying N-terminal peptides in mouse liver specimens. Ortho-phthalaldehyde (OPA) has been employed to block primary amines in proteins, leading to a notable charge disparity at pH 2.7 between terminally blocked peptides and those with free amines following trypsin digestion. This approach confirmed a holistic profiling of 2271 canonical N-termini, and 1650 canonical C-termini, as well as 645 protein neo-N termini from HeLa cells (Figure 11b) [103]. Site-selective termini with unique properties frequently exist in protein mixtures with low abundance, rendering them challenging to detect in high throughput proteomics. Several ways have been proposed to address the difficulties of isolating these terminal peptides. For example, proline at the N-termini can circumvent conjugation by OPA and primary amines in peptides. The subsequent addition of glutaraldehyde mitigated this constraint by confirming the enrichment of N terminal proline via hydrazide stabilization. The quantity of identified peptides with N-terminal proline rose from 1304 to 4039, illustrating an effective approach for addressing low abundance of proteins [104]. The Simplex High-efficiency Undecanal-based N Termini EnRichment (SHUNTER) is developed based on HUNTER. The researchers performed dimethyl labeling of the protein N-terminus using ^13^CD_2_O formaldehyde, carried out the labeling twice, quenched the reaction, and then repurified the sample with SP3 magnetic beads. By reducing the labeling time, this method enhances the efficiency of dimethylation, the purity of N-terminal peptides, and the number of fully identified N-terminal peptides [105]. It successfully identified 4789 endogenous protein N-termini and 514 complement protein N-termini in human plasma samples. The analysis of free N-termini using trimethoxyphenyl phosphonium (TMPP) labeling has been extensively employed to explore newly generated N-termini from proteolytic processes [106]. A refined dual light/heavy TMPP labeling method, referred to doublet N-terminal oriented proteomics (dN-TOP), enhanced the automatic workflow, enabling efficient, rapid, and reliable high-throughput N-terminome analysis (Figure 11c) [107]. Additionally, TMPP in conjunction with StageTip separation techniques has been implemented to delineate the N-terminal initiation sites of annotated encoding genes on *M. tuberculosis* H37Rv [108].

It was noted that trypsin, augmented by 1H-Pyrazole-1-carboxamidine, may elevate the cleavage rate of blocked lysine, hence improving the ionization efficiency of tryptic peptides. The established method, termed Terminal Amine Guanidination of Substrates (TAGS), found 1814 protein N-termini, of which 1012 unique to Saccharomyces cerevisiae (*S. cerevisiae*) (Figure 11d) [109]. Terminal Amine Guanidination of Substrates–Charge Reversal (TAGS-CR) represents the latest technology for N-terminome analysis. In this approach, protein N-terminal α-amines and lysine ε-amines are first guanidinated using 1H-pyrazole-1-carboxamidine hydrochloride. Following reduction, alkylation, and tryptic digestion, unlabeled peptides are sulfated to confer a negative charge, enabling their removal via SCX, while the guanidinated peptides are selectively enriched. By applying TAGS-CR to the study of the *Staphylococcus aureus* V8 protease in human neutrophils, researchers captured 348 substrates, unveiling molecular mechanisms by which *S. aureus* impedes immune responses and promotes infection (Figure 11e) [110]. This strategy enables rapid and efficient resolution of complex proteolytic events.

**Table 1:** Comparison between different terminal labeling methods applied in terminomics.

Labeling Type	Labeling Method	Sample Type	Detection Depth	Key Biological Finding	Ref.
Positive Enrichment	N-CLAP	purified protein, cell lysate, live cells	278 N-CLAP peptides	novel caspase substrates and cleavage sites during apoptosis	[87]
	SATA-based resin-assisted enrichment method	cell lysate	1672 uniquely labeled N-terminal peptides	revealed complex proteolytic processing events in *A. niger*	[88]
	CHOPS	purified protein, cell lysate	thousands of N-terminal peptides	DPP8/9 substrate profiling; cellular N-terminome profiling	[90]
	Subtiligase-mediated biotinylation	cell lysate	906 caspase-3 and 124 caspase-9 substrates	identification of caspase substrates	[94]
	Sortase A-mediated chemoenzymatic enrichment	cell lysate	>2000 unique N-terminal glycine peptides from >1000 proteins	systematically reveals N-terminal glycine as a degradation signal with bidirectional regulatory roles in protein stability	[98]
Negative Enrichment	charge reversal combined with SCX enrichment	cell lysate	over 3000 protein N-termini	loss of cathepsin B/L alters N-terminal processing of proteins	[100]
	OPA labeling-SCX fractionation	cell lysate	2271 protein N-termini; 1650 protein C-termini; 645 neo-N-termini	provides more reliable identification of neo-N-termini	[103]
	TMPP-labeled dN-TOP approach	cell lysate	2714 proteins; 897 N-terminal peptides; 693 unique protein N-termini	identified 302 novel cleavage sites, validated mitochondrial protein processing mechanisms	[107]
	TAGS	cell lysate, live cells	1814 protein N-termini identified in *S. cerevisiae*	discovery of new pyroptosis-related cleavage sites	[109]
	TAGS-CR	cell lysate	346 V8 substrates with 454 unique cleavage events	V8 protease regulates neutrophil functions by cleaving proteins involved in their activity	[110]

## 5. Terminal Modification Applied in C-Terminomics

The instinct homogenous carboxylic acids in aspartate, glutamate, and C-termini make it challenging to specifically label C-termini. Typical trypsin digestion leaving one proton also increases low ionization efficiency for identification. Despite α-carbon epimerization at the C-termini showed its selectivity, its poor conversion still limits broader applications in biological systems. The integration of a bifunctional reagent including biotin and arginine utilized typical oxazolone chemistry and enabled C-terminal enrichment. This de novo C-terminal sequencing resulted the identification of a total of 183 C-terminal peptides from *T. tengcongensis* [47]. Similarly, activated hydrazine derivative conjugated with 2-hydrazino-2-imidazoline to produce a guanidino moiety displaying positive peaks-ion peaks to enhance sensitivity [111]. Incorporating a bromine signature into the C-terminal oxazolone formation also has produce a succession of recognizable y-ions, thus improving detection sensitivity [45]. Besides, positive charges on C-termini by N,N-dimethylethylenediamine (DMEDA), and (4-aminobutyl) guanidine (AG) have proven particularly advantageous in mass detection, including the identification of C-terminal proteome [112,113]. In contrast, cyanogen bromide allows the cleavage for methionine to generate internal termini with homoserine lactone derivative, allowing the identification of C-termini as a singlet in mass detection. This method provided simple and high-throughput sample preparation for the C terminal proteome to study proteolytic processing events [114]. Furthermore, photocatalyzed decarboxylative alkylation (as discussed in Section 3.2) has been applied for C-terminal labeling. This method demonstrated over 50% yield in proteomic digests and shows particular promise for terminal peptide enrichment strategies due to its inherent preference for the C-terminus (Figure 11c) [115]. Different approaches prioritize various aspects of C-terminome analysis, and combining these methods is essential for achieving comprehensive coverage [83,84]. While the C-terminome is often identified through specific protease digestion and removal of internal peptides, direct labeling of C-termini remains an emerging technique. This promises more precise localization and a better understanding of dynamic processes.

The intrinsic chemical properties at C-termini may interfere with site-selective modifications due to the presence of numerous carboxylic acids in the surrounding side chains, despite the structural accessibility advantages of the terminal position. To resolve this, enzymatic engineering for selectively labeling protein C-termini have been explored, including the utilization of carboxypeptidase Y to introduce an affinity tag that ensured the capture and identification of C-terminal peptides from complex cell lysates [73]. Nevertheless, carboxypeptidase Y is still dependent on C-terminal amino acids which may result in bias for terminome identification. Advancements in protease engineering will enable the development of enzymes with distinct cleavage specificities, such as LysC [116], LysargiNase [117]. Those specific proteases generate distinct terminal sites, enabling C-terminal enrichment and subsequent identification. Beyond facilitating site-selective ligation, these synthetic enzymes are capable of recognize internal sequence sites, allowing for varied termini generation. As a result, these advancements in synthetic enzymatic tools hold significant promise for overcoming current challenges in terminome analysis, pushing the boundaries of protein characterization and biological discovery.

## 6. Terminal Modification Applied in Bioconjugation and Therapeutics

Achieving site-specific molecular conjugation on proteins represents a significant extension of selective terminal labeling technology. This advancement not only highlights the crucial role of selective terminal labeling in terminal omics but also demonstrates its considerable potential in other fields. In the development of antibody–drug conjugates (ADCs), researchers have employed *Pyrococcus horikoshii* biotin ligase to conjugate p67 (the 67 amino acid carboxyl-terminal domain of human propionyl-CoA carboxylase α subunit) to the C-terminus of the light or heavy chain of trastuzumab, constructing an ADC and validating its activity [118]. A novel “AbClick Pro” linker technology based on Fc-binding peptides has been developed, enabling the highly efficient attachment of monomethyl auristatin E (MMAE) to the highly conserved lys248 site on human IgG1 heavy chains. This approach allows precise modification of the ε-amino group. Using this method, researchers successfully constructed a conjugate between a PD-L1 antibody and MMAE (durvalumab–MMAE) [119]. In the field of radioimmunotherapy, a novel bifunctional chelator, H_2_MacropaSqOEt, has been designed. One end of this molecule selectively reacts with the ε-amino group of lysine (Lys) residues on the antibody surface, while the other end efficiently chelates the therapeutic radionuclide actinium-225. The constructed radioimmunoconjugate exhibited remarkable tumor-targeting capability and therapeutic efficacy in a renal cell carcinoma model [120]. Regarding cellular therapy for tumors, to enhance the antitumor efficacy of γδ T cells, researchers ingeniously utilized metabolic glycoengineering to incorporate non-natural sugars (such as AMS-ManNAz-P) into terminal sialic acids of cell surface glycans. Subsequently, through efficient click chemistry, antibodies targeting tumor antigens (such as PD-L1) were anchored onto the cell surface. This chemical engineering of the cellular “glycan terminus” successfully endowed γδ T cells with tumor-targeting ability, significantly enhancing their cytotoxic efficacy both in vitro and in mouse models [121]. Selective terminal labeling technology has evolved into an effective tool for drug design and the development of tumor treatment strategies. In the future, with the advancement of more diverse bio-orthogonal reactions and enzymatic tools, this technology will continue to provide powerful support for the research of more complex and effective diagnostic and therapeutic methods.

## 7. Challenges and Outlooks

The expanding repertoire of site-selective labeling techniques is propelling terminomics forward, allowing for the exploration of intricate biological systems with exceptional precision. The growing array of selective labeling techniques allows a tag at termini, which can be characteristic mass increase for identification. Additionally, direct labeling at termini enables semi-protease digestion, avoiding bias from typical trypsin cleavage in enriched process. Present labeling techniques improve chemoselectivity at termini and decrease off-target reactions within cellular and extracellular conditions. Nevertheless, the solubility for labeling proteins is required to be considered for the recovery for mass detection. Notwithstanding the introduction of 2-PCA and sublitigase as exemplars, further advancements in labeling kinetics and efficiency will be concentrated on in-depth profiling of the terminome. Furthermore, the specification of parameters in biological contexts, such as pH, reaction concentration and time, continues to be essential for enhancing targeted efficiency while minimizing non-specific conjugation. The instinct of terminomics tends to mind the efficiency of protease digestion, proteins solubility, and the inherent properties of terminal peptides, which significantly affect the depth of mass spectrometry coverage. Although progresses have been made in sample preparation methods to reduce sample complexity, there remains a significant need for improved terminal peptide stability and robust handling systems to effectively manage large, complex proteomic datasets [83]. Techniques, like tandem mass tag (TMT) labeling [122] and isobaric labeling [123], have introduced multiplexing capabilities that generate consistent, reliable data for experimental setup. Moving forward, there is potential to combine multiplexing with real-time quantification strategies to dynamically track terminal dynamics.

Variations of protein expression, protease activities, and proteolysis between samples may compromise terminomic data reproducibility. The issue of biological variations, like cell types and tissues, are required to pretreatment, which add another dimensional complexity for detection and identification. Selective chemical conjugating and enzyme-mediated tagging strategies offer controlled and specific labeling at predetermined sites, reducing non-specific interactions and improving labeling fidelity. Integrating these techniques with innovations in single-cell analysis and microfluidic technologies could further advance terminome beyond bulk profiling, revealing critical variations among cellular subpopulations [124,125]. The evolving techniques for sensitive detection, like sequential windowed acquisition of all theoretical fragment ion mass spectra (SWATH-MS), provides comprehensive fragment ion datasets with high reproducibility, thereby improving data quality in comparative studies [126,127]. Top-down mass spectrometry is also arising for low-abundance or rare protein termini as field asymmetric ion mobility spectrometry (FAIMS) demonstrates efficacy in separating heterogenous peptide mixtures [85,128]. The classic peptide-centric proteomics generates a plethora of data derived from multiple proteins. Despite the enriched collection before MS detection, low-abundance peptides are still obscure from a significant amount of noise. The analysis tools for terminomics like MANTI and TermineR focus on bioinformatics data processing and annotation [129,130,131]. Meanwhile, machine leaning and deep learning are encouraged to strengthen peptide-to-spectrum matching and refine intensities of fragment spectra, improving peptide prediction and identification within large proteome [132]. The continued evolution of labeling approaches, high-resolution MS techniques, and data analysis strategies is likely to broaden the impact of terminome to explore more specific proteoforms and proteolytic processes collating with disease stages or therapeutic responses.

It is noteworthy that different N-terminomics identification methods exhibit complementarity. Ziegler and colleagues revealed that the N-terminal sites identified by FAIMS and bRP fractionation methods show limited overlap. Simultaneously, they also applied enrichment-based N-terminomics leveraging HUNTER to enable the discovery of an additional sites not detected using either FAIMS or bRP [86]. Employing diverse N-terminomics identification methods is beneficial for expanding the coverage of proteomics and constructing a more comprehensive protein map.

Despite major advances in N- and C-terminomics technologies in recent years, several challenges remain. Classical proteomics typically infers protein presence from peptide data rather than directly identifying intact proteins. When peptides are shared among multiple proteins, additional protein grouping and integration are required for comprehensive analysis. Chemical labeling strategies are susceptible to non-specific or incomplete labeling. The intrinsic nature of chemical labeling often neutralizes or sterically hinders basic residues, compromising the efficiency of standard protease digestion. This leads to intractable peptides that negatively affect MS coverage depth. Additionally, bulky terminal tags can alter the fragmentation patterns of peptides (e.g., inhibiting b/y-ion generation), complicating precise site localization and increasing peptide-to-spectrum match (PSM) ambiguity. These issues can be mitigated, to some extent, by using multiple biological and technical replicates and by employing different proteases to generate N-terminal fragments of varying lengths, thereby increasing the identification frequency of specific N-terminal peptides. Nonetheless, there remains a clear need for improved methods that simplify enrichment procedures, enhance throughput, and increase analytical sensitivity. Developing integrated strategies that jointly analyze N-terminal and C-terminal neo-peptides will enable more precise mapping of cleavage site specificities at higher resolution, offering significant research value.

## 8. Conclusions

This review outlines advanced techniques for selective labeling at termini, emphasizing the development of chemical techniques and enzymatic methods for site-selective modification in proteins and peptides. Nevertheless, no one-fits-all method is applied for all circumstances. These reported approaches were utilized with specific enhancements in selectivity, efficiency, and compatibility. While we applied these techniques in biological contexts, multidimensional factors are required to be considered. The complicated circumstance of biological samples involves minimal disturbance for dynamic process and accurate labeling sites. The termini of proteins are not merely passive structural components but play an essential role in the biological activity of proteins. Thus, the leverage for terminal labeling needs careful procedures to snapshot fine distinction in cellular pathways. To explore the proteome web of protein termini, selective labeling has been utilized in the workflow to detect and identify the spectrum. Those applications in terminomics have illuminated transcriptional and translational regulations, alternative splicing, and proteolytic processing, elucidating the intricate networks of protein interactions. Direct site labeling streamlines the enrichment process for detection, yet the main challenge lies in the in-depth labeling of targeted proteins that exhibit diverse structures and stabilities. It will be expected to enable the operation of various proteins simultaneously at their terminus, expanding applications in live cells, tissues, and beyond. Addressing selective labeling techniques will enable streamlined and effective processes for terminomic analysis, shedding light on the annotation for proteome web.

## Data Availability

No new data were created or analyzed in this study. Data sharing is not applicable to this article.
